# O.2.2-10 Preventing and managing injuries in social sport programs

**DOI:** 10.1093/eurpub/ckad133.125

**Published:** 2023-09-11

**Authors:** Kiera Staley, Alex Donaldson, Erica Randle, Matthew Nicholson, Andrea Bruder, Paul O'Halloran

**Affiliations:** La Trobe University, Australia; La Trobe University, Australia; La Trobe University, Australia; Monash University Malaysia; k.staley@latrobe.edu.au; La Trobe University, Australia; La Trobe University, Australia

## Abstract

**Purpose:**

Safety is critical to participant recruitment and retention in social sport programs targeting insufficiently active (IA) people as their lack of fitness and limited previous exposure to physical activity likely places them at a higher risk of injury. This study sought to understand the injury prevention/management qualifications, knowledge, confidence and practices of program deliverers (coaches or instructors) across four social sport/active recreation programs targeting insufficiently active women.

**Methods:**

Between 2021 and 2022, we sent an online survey via text message to 83 program deliverers in Victoria, Australia after they had delivered their social sport/active recreation programs to 1674 insufficiently active women. Delivers were asked their: age and gender; years delivering sport/physical activity programs targeting insufficiently active adults; injury prevention/management qualifications; and what injury prevention/management measures they undertook during programs. They also rated their knowledge of injury prevention/management measures (0 = none; 10 = comprehensive); and their confidence incorporating such measures into a program (0 = none; 10 = extremely). We analysed data using descriptive statistics.

**Results:**

Sixty-eight program deliverers (response rate 82%) completed the deliverer survey. Their experience in delivering programs to insufficiently active participants was limited (62% < two years) but nearly all (90%) reported providing some form of injury prevention/management advice to program participants — warm up or cool down, activity recovery, skill or technique, and/or corrective footwear were most common, but the type of advice provided differed across the programs. Forty percent reported no formal injury prevention/management qualifications. Their mean self-rated knowledge of injury prevention/management measures was 5.7 out of 10 and their mean confidence rating for incorporating such measures into programs was 6.0. Nearly half (46%) reported low confidence (≤5 out of 10;).

**Conclusions:**

While most program deliverers lacked formal qualifications, had limited injury prevention/management knowledge and/or low confidence, they provided some form of injury prevention/management advice to participants. Program delivers would benefit from increased injury prevention/management knowledge, capacity and/or capability to assist with the program experience, reduce injury risk for IA participants, and hopefully improve deliverer confidence. Advice given by deliverers with limited knowledge and experience should be monitored.

**Support/Funding Source:**

Victorian Health Promotion Foundation (VicHealth).

